# Upgrading and Enhancement of Recycled Polyethylene Terephthalate with Chain Extenders: In-Depth Material Characterization

**DOI:** 10.1021/acs.iecr.4c00018

**Published:** 2024-07-05

**Authors:** Christian W. Karl, Bjørnar Arstad, Madina Shamsuyeva, Jacek Lecinski, Kjell Olafsen, Åge Gellein Larsen, Stephan Kubowicz, James Comerford, Hans-Josef Endres

**Affiliations:** † SINTEF Materials and Nanotechnology, Polymer and Composite Materials Group, P.O. Box 124 Blindern, 0314 Oslo, Norway; ‡ SINTEF Process Technology, Process Chemistry and Functional Materials Group, P.O. Box 124 Blindern, 0314 Oslo, Norway; § IKK—Institute of Plastics and Circular Economy, Leibniz Universität Hannover, An der Universität 2, 30823 Garbsen, Germany

## Abstract

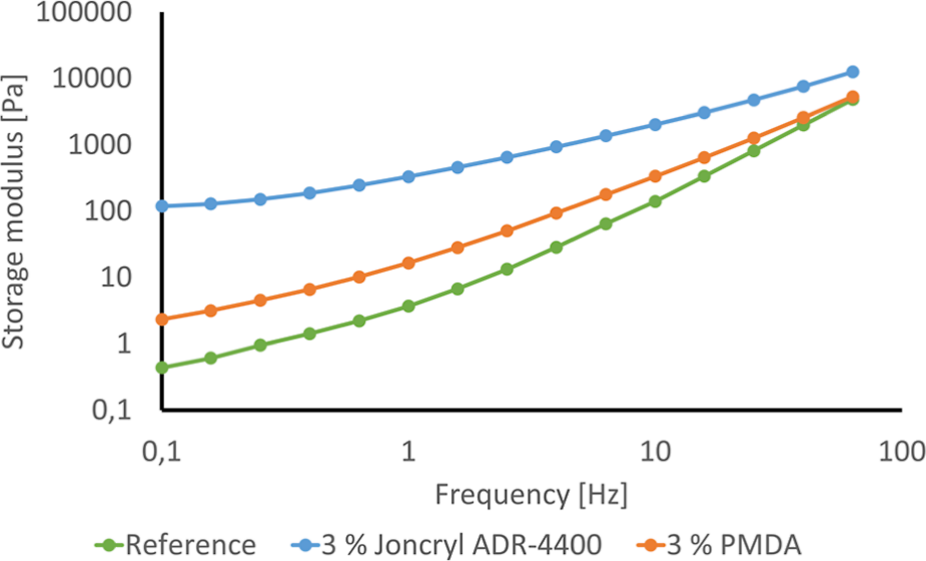

Chemical chain extenders (CEs) can be used to restore the properties of recycled low-molecular-weight polyethylene terephthalate (PET). The aim of this work is to investigate the influence of the type and concentration of the CEs Joncryl and pyromellitic dianhydride (PMDA) on the viscosity and other rheological properties with a unique combination of different methods based on industrial samples originating from recycled PET bottles and trays. The resulting chain-extended thermoplastics were characterized by a combination of differential scanning calorimetry, viscometry, cone plate rheometry, pyrolysis—gas chromatography—mass spectroscopy, optical photothermal infrared spectroscopy, ^13^C solid-state- and ^1^H NMR liquid spectroscopy, and size exclusion chromatography. For a recycled PET mixture containing bottle and tray materials, our investigations have shown that a significantly better effect for chain elongation can be achieved with Joncryl compared to PMDA. This can presumably be attributed to water molecules formed during the use of PMDA, which accelerate the degradation of PET. The storage modulus values are therefore significantly higher for the samples with Joncryl compared to PMDA. The results of this study show that chain extension with Joncryl proceeds better compared to the reaction with PMDA.

## 1 Introduction

With a global production amount of 24,22 Mt in 2021,^1^ polyethylene terephthalate (PET) is one of the most common polymers in the plastic industry, and PET-based packaging products are extensively recycled. Depending on the country, the amount of PET recycled varies considerably. Norway, for instance, belongs to the leading countries in the world with respect to PET recycling with a recycling rate of 97% for plastic bottles. In the European Union, on the other hand, only about 58% of plastic bottles were recycled on average in 2018.^2^ An inventory of the Norwegian market has recently shown that different actors in the value chains have different views on the amount of recycled PET pellets (rPET) that can be included in the material flow before the quality deteriorates. Furthermore, it seems that the assessment of the available technology is situation-dependent.^3^ PET is strong, impact-resistant, and is widely used in beverages, food, and other liquid container applications as well as in engineering resins, often in combination with glass fibers. Recycling processes are the best way to economically reduce PET waste, and different routes for recycling can be applied.^4,5^ The recycling of PET can be divided into two main process methods of plastic recycling: chemical recycling and mechanical recycling.^6,7^ During chemical recycling, the polymer is converted into monomers or oligomers through chemical reactions.^8^ The chemical reactions for depolymerizing PET include hydrolysis (neutral, acidic, alkaline, or enzymatic), alcoholysis (e.g., methanol or glycol), and aminolysis.^9,10^ Chemical recycling of PET is at an earlier stage of development than mechanical recycling, as most existing technologies are at a pilot stage. However, glycolysis appears promising for large-scale production.^7^

The mechanical recycling of PET by melt reprocessing is the most used since it is relatively simple, requires low investments, utilizes established equipment, is flexible in terms of feedstock volume, and has little adverse environmental impact. Improvement of the quality of recycled PET can be achieved by various additives.^11^ Many studies have been carried out with the use of chain extenders (CEs) to recover the properties of low-molecular-weight PET, and experiments with different CEs used in the chain extension of PET have been reported in the literature,^12−15^ including Joncryl which has been used in many polymers for chain extension,^12,13,17^ and pyromellitic dianhydride (PMDA)^16,18^ which has shown how molecular weight can be increased, and crystallinity decreased with its incorporation. PMDA has proven to be an effective chain extender in a reactive extrusion system for recycled PET on an industrial scale.^18,19^ Reactive extruded recycled PET was obtained with various carboxyl contents compared to nonextended recycled PET. The reactive extrusion system was successfully used in an existing normal extrusion system with appropriate adaptation to the extruder temperatures and screw speed.

**Figure 1:**
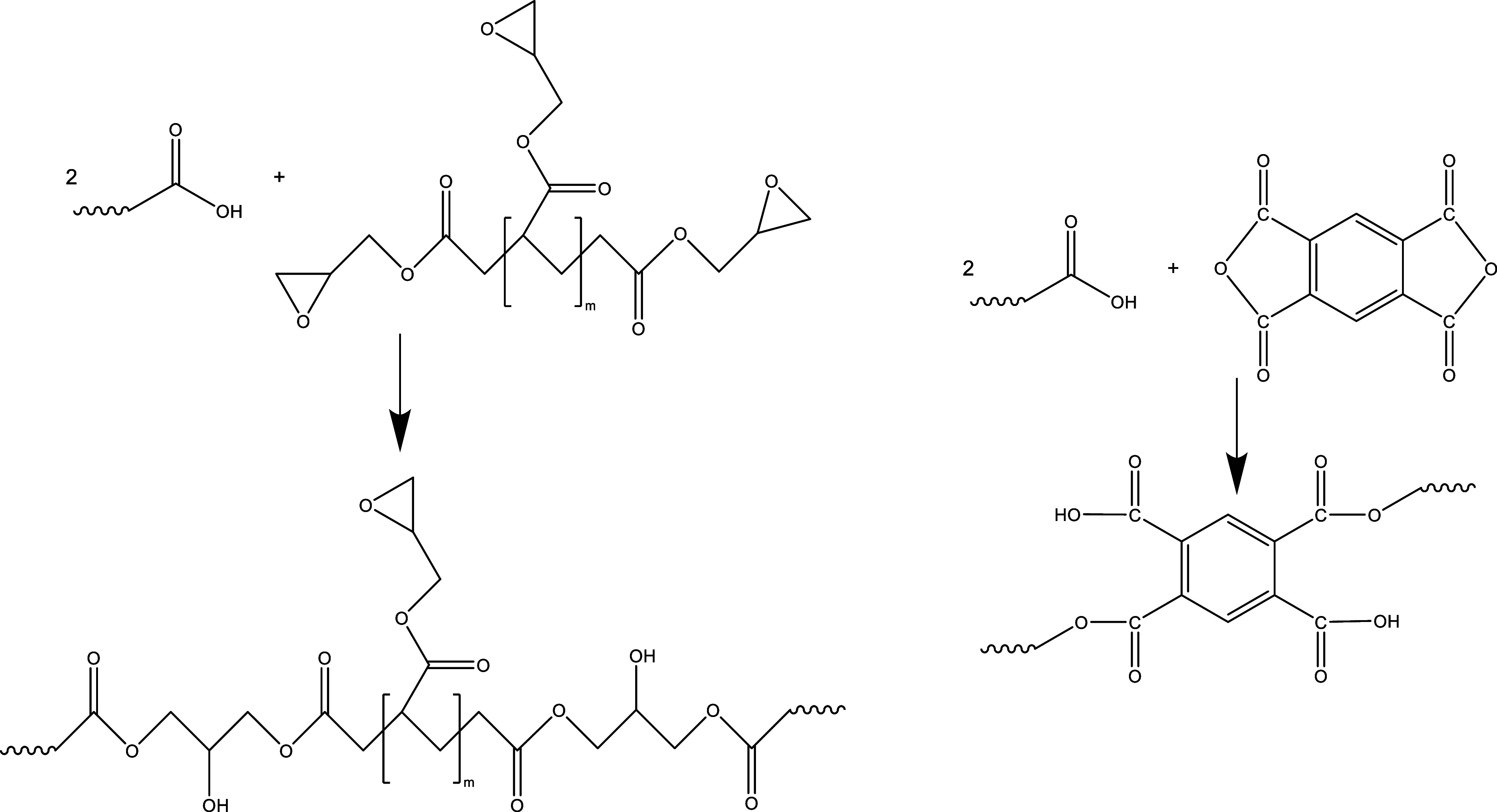
Scheme of the chain extending reaction of PET by a multifunctional epoxydic oligomeric additive (Joncryl), left, and PMDA, right.

During the melt reprocessing of PET, the polymer undergoes chemical, mechanical, thermal, and oxidative degradation that reduces its molar mass and its viscosity, melt strength, and mechanical properties. Especially at very high process temperatures (around 300 °C), the rate of these degradation reactions is extremely rapid. Hence, it limits the usefulness of recycled materials for many applications. When CEs such as Joncryl or PMDA are added, increased molar mass can be seen and is attributed to the reaction of epoxy groups with the carboxyl (and to a lesser extent, hydroxyl) ends of PET fragments, resulting in the combination of two or more fragments and the extension of the PET chain, as schematically shown in Figure 1.^12^

The reaction between the chain extender PMDA and the recycled PET can be divided into three possible types:^18^ branching, blocking, and coupling.

Branching reactions, in which PMDA molecules react with more than two PET molecules and/or more than one PMDA molecule with more than two PET molecules, lead to cross-linking and finally to gel formation. Blocking and coupling reaction rates are important determinants for increasing the molar mass or viscosity of recycled PET in the extruder. In the mechanism of the chain extension reaction, the PET hydroxyl end group attacks the anhydride group of PMDA. This leads to a coupling between PET and PMDA, i.e., the formation of two carboxyl groups. The resulting carboxyl groups will later react with PET and introduce more coupling and/or branching reactions and produce a water molecule at each stage of the reaction. The chain extender PMDA is tetrafunctional, which means that one molecule of PMDA can link four PET chains. A typical reaction that could take place would be a complete combination of the four functional PMDA groups with four PET molecules to form two water molecules.

The main factors that determine the blocking, coupling, and branching reactions, and thus the carboxyl content, are the PMDA concentration and reaction time. Impurities, such as moisture and polyvinyl chloride, will increase the PET degradation reaction rates^20,21^ and the carboxyl content. The carboxyl content of recycled PET has negative effects on the physical properties of the material and hydrolytic stability as discussed by Awaja and coauthors.^18^

In the present work, we have investigated the chain extension reaction of recycled PET from a mix of bottles and trays using two different common CEs (Joncryl and PMDA) to obtain a detailed analysis of the resulting products. The force development in a Midi2000 batch extruder during compounding has shown the timescale for the reactions. The resulting chain-extended thermoplastics were characterized for the first time by a unique combination of the following methods: differential scanning calorimetry (DSC), viscosity, cone plate rheometry, pyrolysis GC–MS, optical photothermal IR, ^1^H NMR of dissolved samples, ^1^H–^13^C cross-polarization solid-state NMR spectroscopy, and size exclusion chromatography (SEC) measurements. SEC investigations have been used to estimate relative molar mass changes during processing that may be attributed to degradation or chain extension.

## 2 Experimental Section

The recycled PET was compounded with different CEs in various weight percentages, and the force curves [*F*(*t*)] were recorded from the compounding in a mini-batch extruder (Midi 2000, DSM) during extrusion. The materials were analyzed by the following characterization techniques: DSC (enthalpy and crystallinity), viscosity, rheology, ^1^H NMR spectroscopy, ^1^H–^13^C CP NMR spectroscopy and SEC, pyrolysis–gas chromatography–mass spectroscopy (pyrolysis GC–MS), and optical photothermal IR analysis.

### 2.1 Materials

Recycled PET was obtained from Armacell (Armacell Benelux S.C.S., Belgium) in the form of flakes and was used without blending with other PET types. The recycled PET was a mix of 80% bottles and 20% trays from German yellow bin collection; the PET fraction was sorted for clear material only. Bottle-grade PET contained comonomers in concentrations of 1–2% of each IPA (isophthalic acid) and DEG (diethylene glycol). The comonomers IPA and DEG are added to reduce the rate of crystallization during processing. Crystallization is undesirable as it leads to the formation of large crystallites which reduce the clarity of the product.^22,23^

The following two CEs were used: pyromellitic dianhydride (PMDA, Sigma-Aldrich) and Joncryl, ADR-4400 (BASF). The CEs are designed to rebuild the molecular weight of degraded condensation polymers such as PET. The low polydispersity and the tailor-made functionality of the epoxy groups have demonstrated very good properties in nonlinear chain extension.^14^ The information about the different Joncryl CEs used in this work is described elsewhere.^12^ ADR-4400 has a molecular weight (Mw) of 7100 g/mol.^17^

### 2.2 Material Compounding

The PET flakes were dried at 140 °C for 17 h, and the CEs were dried at 120 °C for 3 h before use to minimize hydrolysis and decomposition. PET batches with different amounts (in wt %) of CEs were compounded in a mini-batch extruder Midi2000 (DSM) for 10 min, with the chamber walls kept at 280 °C. The extruder is equipped with screws with an enhanced feeding zone. The extruder barrel was flushed with N_2_ during compounding. The rotation speed was set to 70 rpm. The pressure built up along the screw was balanced by the force on the extruder barrel at the bottom, which was recorded as a function of time. Under stable flow conditions, this force will depend on the melt viscosity and was stored as a function of time—*F*(*t*). Comparative concentrations of the chain extender were chosen according to the recommendation of the manufacturer and the literature.^15^ All prepared samples are summarized in Table 1.

**Table 1:** Processed Sample Grades Included in the Study

sample number	weight % (%)		chain extender type
	recycled PET	chain extender (CE)	
1	100	0	none
2	99.5	0.5	Joncryl ADR-4400
3	98.5	1.5	Joncryl ADR-4400
4	97	3	Joncryl ADR-4400
6	97	3	PMDA

### 2.3 Characterization

#### 2.3.1 Differential Scanning Calorimetry

The samples were analyzed with a TA Instruments DSC 2500 system. The heating rate was 10 °C/min from room temperature to 350 °C, followed by an isothermal step for 5 min at 350 °C. Thereafter, the sample was cooled to −20 °C with a rate of 10 °C/min, followed by second heating to 350 °C with a rate of 10 °C/min. Sample weights were from 3.5 to 5.7 mg. The degree of crystallinity (Xc) was calculated using the melting enthalpies recorded from the second heating^16^ using the following equation
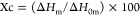
where Δ*H*_m_ is the crystalline melting enthalpy and Δ*H*_0m_ is the theoretical melting enthalpy for a 100% crystalline PET (140 J/g).^16^

### 2.4 Solution Viscosity

Inherent viscosity was measured at 30.0 °C on 5 g/L solutions of PET in 60/40 wt % mixture of phenol/1,1,2,2-tetrachloroethane. The method is based on ASTM D4603-18, but instead of a Cannon Ubbelohde viscosimeter, an Anton Paar Physica MCR 300 viscosimeter was used with a cone plate geometry (diameter: 50 mm and cone angle: 1.004°). The measurements were performed with the shear rate from 100 to 600 1/s.

### 2.5 Melt Rheology

The rheology measurements were performed with an Anton Paar MCR-502 rheometer using a 25 mm parallel plate configuration with 1 mm gap. The measurements were performed at frequencies from 0.1 to 100 Hz at a temperature of 265 °C as used by Kruse.^24^

### 2.6 Size Exclusion Chromatography

Molar mass and molar mass distribution were determined by SEC with RI and a UV 280 nm detector. Chloroform/phenol (1/1 vol %) solution was used as a solvent for the samples and chloroform as an eluent. PMMA served as the calibration standard. Gel content was measured according to ISO 10147:2012 using a chloroform/phenol (2:1 vol %) solution.

### 2.7 Pyrolysis GC–MS

Pyrolysis–gas chromatography–mass spectroscopy (Py–GC–MS) was performed with a double-shot pyrolyzer EGA/PY-3030D (Frontier Laboratories Ltd., F-Lab) attached to a Trace 1310 GC and ISQ 7000 single-quadrupole MS system from Thermo Fisher Scientific.

Unaltered ground samples of about 200–450 μg were placed in small crucibles and introduced into the furnace via an autosampler (AS-1020E, F-Lab). Pyrolysis was performed at 600 °C, thermodesorption in the 40–280 °C range at a 20 K/min ramp. The evolved gases were then directly injected into the GC/MS system for analysis. The gas chromatograph was equipped with a low-to-mid-polarity Ultra Alloy capillary column (UA + -5, F-Lab) of 30 m × 250 μm × 0.25 μm film thickness. The carrier gas was helium at a controlled flow of 1 mL/min. In the mass spectrometer, the total ion current (TIC) chromatograms were acquired at 70 eV ionizing energy. The obtained mass chromatograms of substances were analyzed and compared with NIST and F-Search (F-Lab) databases.

### 2.8 Optical Photothermal IR Microscopy

The measurement was performed using a mIRage optical photothermal infrared (O-PTIR) microscope (Photothermal Spectroscopy Corp. Santa Barbara, USA). For data acquisition and processing, the PTIR software (vers. 4.3.7478) of the same manufacturer was used at approximately 30 different locations of each sample. The results represent the average spectrum of measurements made on each sample.

O-PTIR works on the principle of photothermal detection in which an IR quantum cascade laser (QCL 532 nm^–1^) excites the molecular vibrations of the sample in the spectral range of 1800–800 cm^–1^. The detection scheme of O-PTIR includes three main steps. First, a pulsed QCL produces photothermal effects on the sample. Second, the absorption of the radiation is followed by a small local heating, leading to a thermal expansion and a change of the refractive index, and finally a visible probe laser is focused on the specimen, which detects these photothermal IR effects.^25^ O-PTIR is performed in reflection mode, so that the specimen is not damaged. In comparison with the well-known FTIR, O-PTIR enables the acquisition of IR spectrum at a submicron resolution, thus providing more detailed information.

### 2.9 NMR Spectroscopy

^13^C solid-state NMR spectra of solid polymer samples were acquired using ^1^H–^13^C cross-polarization (^1^H–^13^C CP) and a Bruker Avance III spectrometer operating at a magnetic field of 11.74 T. A 4.0 mm double-resonance magic angle spin (MAS) probe head was used at room temperature with a MAS rate of 12 kHz. The spectra were acquired using 2h000 scans, a recycle delay of 5 s, and a Hartmann–Hahn contact time of 2000 μs. Before the Fourier transform of the averaged signals [free induction decays (FID)], zero filling and apodization were applied to improve line shape definitions and the signal-to-noise ratio. The chemical shifts were referenced to tetramethylsilane (TMS) by the substitution method^26^ by setting the high-frequency peak of adamantane to 38.48 ppm.

The ^1^H spectra of dissolved polymer samples were recorded with a Bruker Avance III spectrometer equipped with a BBFO-Plus probe at 298 K, at a magnetic field of 9.4 T (400 MHz proton resonance frequency). Before analyses, the samples were dissolved in a deuterated trifluoroacetic acid-in-chloroform (TFA-d1–CDCl_3_) mix (1:4 w/w), and the chemical shifts were referenced to TMS (included in the CDCl_3_ solvent). The solids dissolved readily, and a transparent solution was observed within an hour. The recycle delay was set to 10 s, and each spectrum is based on 16 accumulated scans (FIDs).

All accumulated FIDs were zero-filled and apodized with an exponential function for improved resolution before Fourier transformation. All NMR spectra were then adjusted by proper signal phasing and baseline corrections. Processing and plotting of all spectra were performed using the software MestReNova v 14.1.1-24571 from 2019.

## 3 Results and Discussion

**Figure 2:**
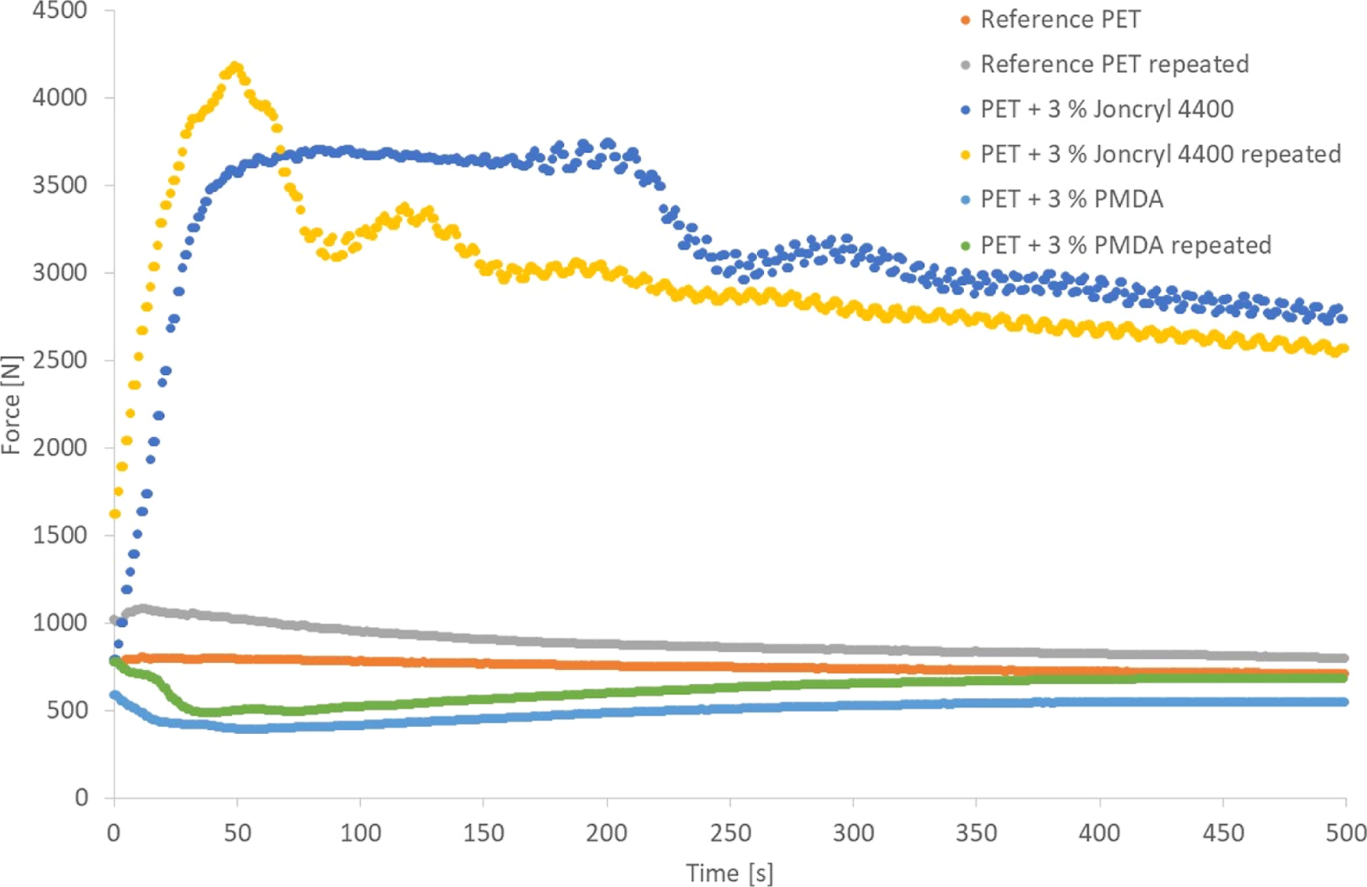
Force vs time curves for the processing of neat PET and PET modified with the CEs Joncryl ADR-4400 and PMDA (duplicate test).

Figure 2 shows force versus time curves measured during compounding for duplicate tests, for which 3 wt % chain extender Joncryl ADR-4400 and 3 wt % PMDA were incorporated. For PMDA as CE, the force versus time curves are about on the same level as unmodified PET. For Joncryl ADR-4400, the force is on a significantly higher level than for unmodified PET and PET with PMDA as CE. The force versus time curves received from the instrument were used for the estimation of the combined effect of degradation and chain extension during processing.^27^ The tests without CE were also performed for comparison. The reproducibility during this process was acceptable, and similar results were shown previously.^27^ For PMDA, a decrease in force versus time could be seen at the start of the extrusion, while the force increases with further extrusion time. This indicates that a degradation of the polymer chains takes place in the beginning while chain extension occurs with further extrusion time, but the chain extension reaction is not sufficient to increase the molecular weight as compared with unmodified PET. The course of the curve for Joncryl could be attributable to the strong overcompensation of the polymer degradation. Tavares et al. have shown that doubling the Joncryl concentration from 1.5 to 3 wt % leads to a 20% increase of the molar mass.^27^ Duarte and Costa demonstrated that the use of Joncryl compounded as CE in a laboratory internal mixer appears to compensate for degradation during processing. At the same time, the molar mass of the polymer could be increased.^15,28^ In Section 3.2 the inherent viscosity and the force from the *F*(*t*) curves are discussed.

### 3.1 Differential Scanning Calorimetry

**Figure 3:**
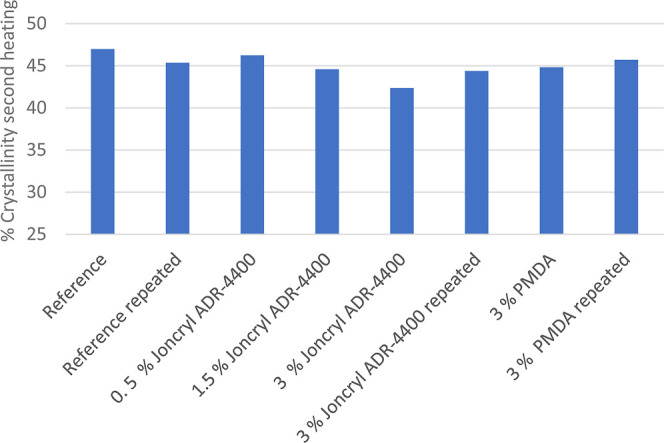
Percentage crystallinity (second heating) for PET with and without added chain extenders.

An example of a DSC thermogram for PET with a chain extender (3 wt % Joncryl ADR-4400) is shown in Figure S1, with crystallization data from the whole data series shown in Figure 3. The blue curve represents the first heating and shows a glass-transition temperature (*T*_g_) of 77 °C, a peak representing cold crystallization at 121 °C, and a crystalline melting peak at around 246 °C. The orange curve represents cooling down and shows a crystallization peak at 203 °C. The gray curve represents the second heating where the cold crystallization peak has disappeared, while the crystalline melting peak is still at 246 °C. Regarding *T*_g_, after reaction with CEs, *T*_g_ was on the same level or slightly higher as compared to pure PET in all cases.

From the DSC data, a slight decrease in crystallinity for the highest concentrations of the chain extender can be derived for samples with Joncryl ADR-4400 as compared with the unmodified PET. The crystallinity of PET has been reported to decrease with the increasing chain extender concentration due to the increased molecular weight and branching^16^ or to increase with the increasing chain extender concentration.^29^ In the case of the observed increasing crystallinity with increasing chain extender concentration, triphenyl phosphite was used as the chain extender. With this chain extender, small molecules are also formed during the chain extension reaction (reaction residues). It was claimed that a higher chain extender concentration also gives more reaction residues that give a plasticizing effect, resulting in more chain mobility which promotes crystallization. However, in the present case, a slight decrease in crystallinity with the highest amount of Joncryl ADR-4400 could be observed which fits with the increased molecular weight and branching.^16^ For samples with 3 wt % PMDA, the crystallinity was on the same level as for unmodified PET. For the 3 wt % samples and PET standard, the reproducibility was tested with a new batch of produced samples, and the same trends as for the first batch were received.

### 3.2 Viscosity and Force Measurements

**Figure 4:**
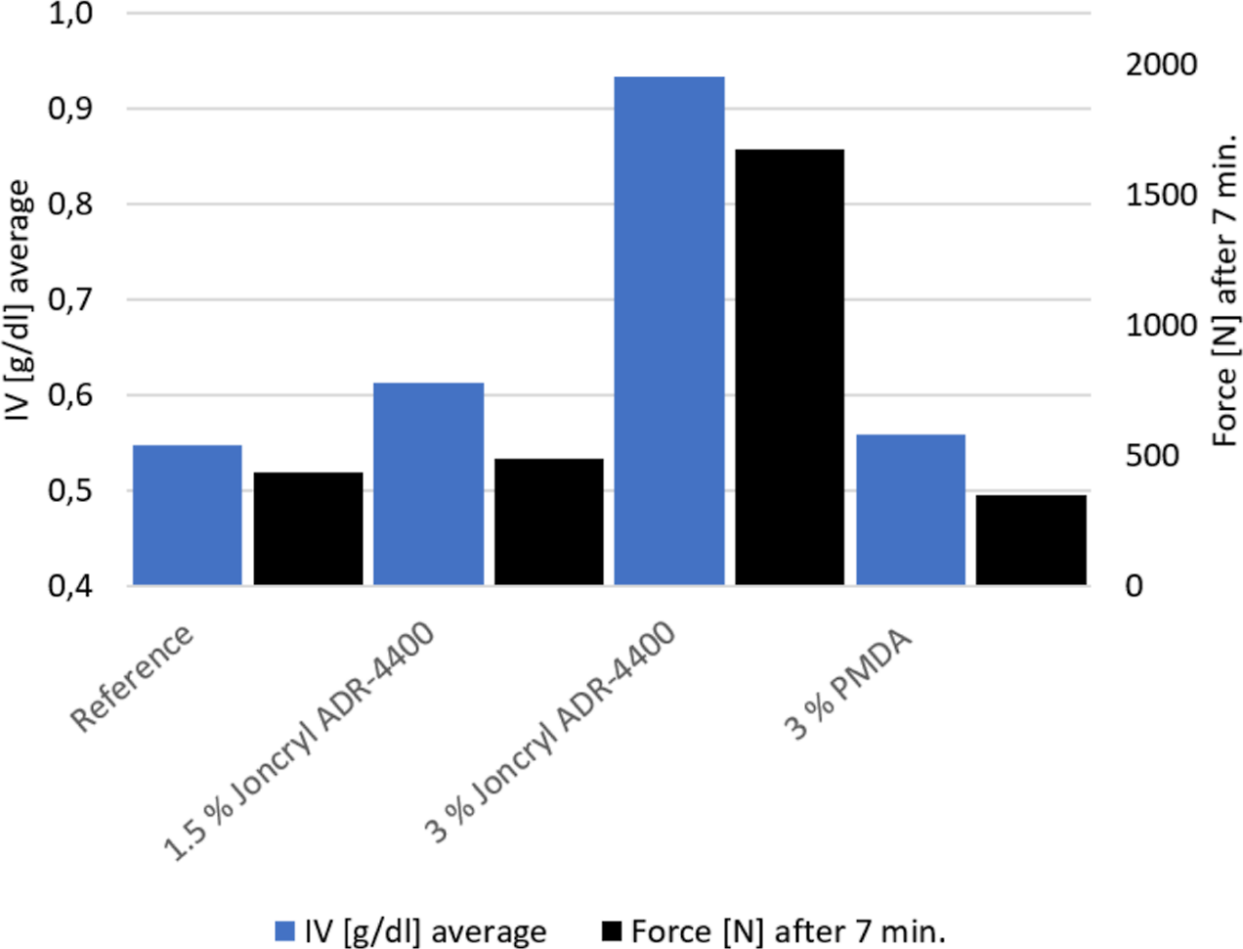
Inherent viscosity and force after 7 min extrusion for unmodified and modified PET with different CEs.

Figure 4 shows a comparison between the inherent viscosity and the force read from the *F*(*t*) curve from extrusion after 7 min. The total extrusion time was 10 min, and the force after 7 min was chosen because after this time the *F*(*t*) curves are quite stable after the CEs have had time to react with PET. In general, there is a very good correlation between the force measured after 7 min during extrusion and the measured inherent viscosity.

Härth et al.^31^ describes the molecular structure of PET modified with PMDA and Joncryl ADR-4368. PMDA has four functional groups that can contribute to chain extension, while Joncryl ADR-4368 has an average of nine functional groups. Joncryl ADR-4400 used in the present case assumingly also has a higher functionality than PMDA. As seen in Figure 4, chain extension with Joncryl ADR-4400 gives an increase in force measured during extrusion and inherent viscosity as compared to the chain extension with PMDA. This can be explained with the higher functionality of Joncryl ADR-4400 giving more long-chain branched structures.

### 3.3 Size Exclusion Chromatography

Table 2 shows the results for SEC measurements and gel content.

**Table 2:** Results of the SEC Measurements and Gel Content

sample	polymer *M*_n_ (g/mol)	low-molecular-weight compounds *M*_n_ (g/mol)	polymer *M*_w_ (g/mol)	low-molecular-weight compounds *M*_w_ (g/mol)	dispersity *D̵*	dispersity of low-molecular-weight compounds *D̵*_LMC_	gel content
reference	34,300	90	80,000	190	2.3	2.2	0 ± 0
0.5% Joncryl ADR 4400	34,400	890	72,700	970	2.1	1.1	0 ± 0
1.5% Joncryl ADR 4400	34,800	960	86,600	1000	2.5	1.1	2 ± 1
3% Joncryl ADR 4400	34,600	60	81,900	710	2.4	12.0	32 ± 1
3% PMDA	11,700	90	45,200	210	3.9	2.4	2 ± 1

The results show that the analyzed samples include the components of different molecular weight distributions. For Joncryl, a partial gelation occurs, and the gel content distinctly increases with the growing concentration. This finding is consistent with the published data from the literature^30,31^ from Härth et al. Since the polymer solution is filtered before the molecular characterization by SEC, the gelled molecules are retained, and the molar mass distributions and the molecular data present the properties of the soluble amount of the samples. Both the weight-average molar mass and molar mass dispersity run through a maximum above 1.5 wt % chain extender concentration. Similar to that remarked by Härth et al.,^30,31^ the gel formation can be considered as the reason for the decrease of *M*_w_ after the maximum due to the prevalent reaction of longer molecules between each other, which leads to the formation of a gel. Although the change of number-average molecular mass of polymer chains is not pronounced strongly, as *M*_n_ remains virtually identical for all Joncryl samples tested, the change of mass average *M*_w_ is visible, which shows that the polymer has undergone reactions leading to chain extension. Moreover, the detected small molecular moieties dramatically change their molecular mass due to the introduction of a Joncryl chain extender. Thus, it is most likely that the detected contamination in the form of small-molecular moieties reacts more readily with the Joncryl additive than the additive itself with PET chains, due to the superior mobility of the former in the solution. Joncryl consumed primarily by contaminants remains unavailable to the PET chains until the additive is added in excess or the concentration of the contaminants runs low. This conclusion is corroborated by the gel content measurements, which show that for lower chain extender concentrations of 0.5 and 1.5 wt %, the polymer remained in solution, max. 2% of the sample was cross-linked and formed a gel structure, whereas at 3 wt % Joncryl concentration, the gel content increased to around 32%. Molar mass dispersity (*M*_w_/*M*_n_ ratio) increasing up to 1.5 wt % of Joncryl content confirms the hypothesis of the achieved chain extension described above. A certain deviation from the literature values is due to the differences between the PET samples analyzed and the fact that in this study PET samples from diverse sources were investigated. Above the saturation with the Joncryl chain extender (somewhere between 1.5 and 3%), the polydispersity decreases as well. The PD value of small-molecular moieties in 3% Joncryl sample is significantly higher than that in other tested samples. This also suggests that most of small molecules present in the solution have participated in a reaction with the chain extender.

### 3.4 Pyrolysis GC–MS

The pyrolysis- and thermodesorption-GC/MS results of PET samples with Joncryl ADR 4400 additive are presented in the following figures. The results show peaks, which correspond to the substances detected and identified in the mass spectrometer depending on their retention time in the gas chromatography column. All but one of the substances detected in the pyrolysis modus are characteristic for the PET matrix and include benzoic acid and its derivatives and derivatives of terephthalic acid. The relative TIC areas of those peaks remain approximately constant, apart from the peak appearing at a 6 min retention time mark, which can be ascribed with high certainty to styrene. The intensity of this peak increases in samples along with the increasing Joncryl additive concentration, as can be seen in Figure S2.

**Figure 5:**
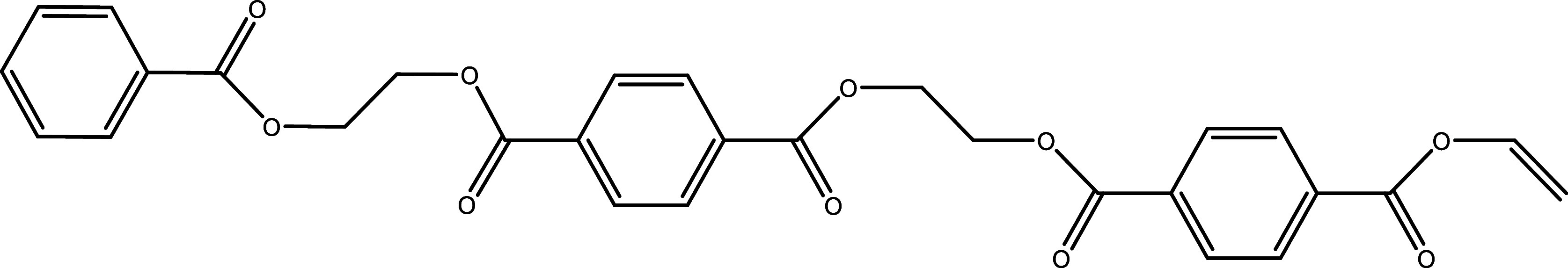
Molecular structure of a quasi-PET trimer or 2-(5-((2-(benzoyloxy)ethoxy)carbonyl)benzoyloxy)ethyl vinyl terephthalate.

The results of the measurement in thermodesorption mode also show the presence of styrene but only at higher concentrations, i.e., 1.5 and 3.0 wt %. Furthermore, in the samples with 1.5 and 3.0% of CE, a peak at 15.5 min was observed, which is assigned as the styrene trimer (see Figure S3). In addition to that, a peak at 18.8 min is observed in all samples containing Joncryl ADR 4400. This signal can be identified as a quasi-PET trimer or 2-(5-((2-(benzoyloxy)ethoxy)carbonyl)benzoyloxy)ethyl vinyl terephthalate (see Figure 5). Apparently, the presence of this substance can be attributed to the addition of CEs as detected.

In the case of the samples with PDMA, a pyromellitic anhydride was observed both in the reference sample and in the sample containing PMDA (see Figure S4). In conclusion, the results of Py–GC/MS show that Joncryl ADR 4400 provides a characteristic fingerprint by the styrene peak in pyrolysis and thermodesorption. Thus, the styrene peak could be used to quantify the amount of chain extender. However, it only appears at higher concentrations. The presence of PMDA in the PMDA sample can also be uniquely identified and quantified in thermodesorption mode.

### 3.5 Optical Photothermal IR Spectroscopy

Figure S5 shows a typical O-PTIR spectrum of a reference PET without an added chain extender, as reported in the literature.^25^ The assignment of the identified bands according to the literature is summarized in Table 3. Slight deviations of the wavenumbers compared with the literature are due to the differences of the measurement systems and the analyzed PET charges.

**Table 3:** Assignment of the Identified Bands According to the Literature

O-PTIR	
absorption bands (cm^–1^)	functional group
1727	C=O^25,32,33^
1341, 1408	bending and wagging vibrational modes of the ethylene glycol segment^32,34^
1121, 1248–1285	C(O)–O in the ester group^25^
1101	methylene group^32,34^
1019	ring stretching^35^
874	bending and wagging of CH in the aromatic ring^32,36^

**Figure 6:**
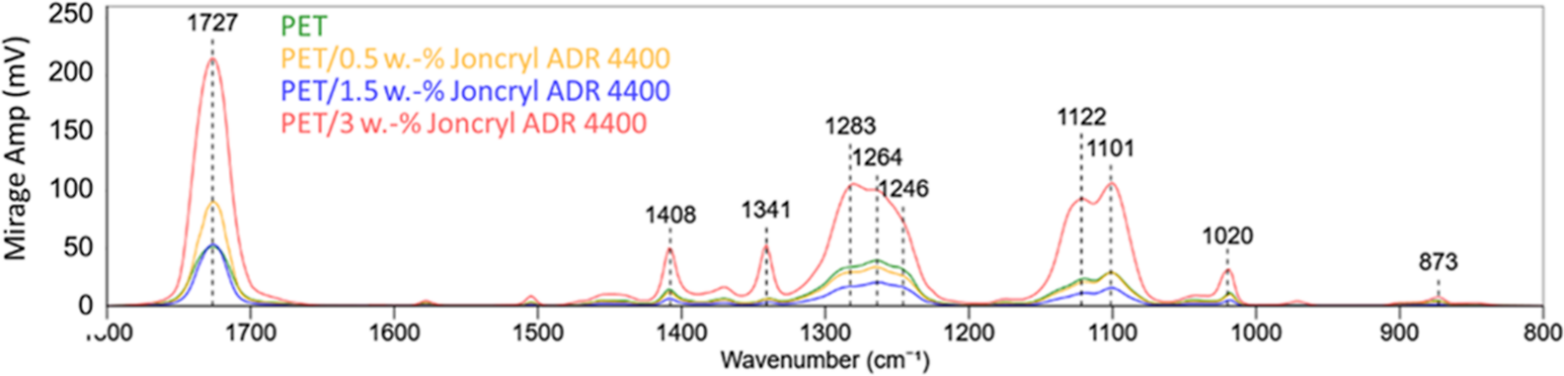
Spectra of the reference sample and samples including Joncryl ADR 4400.

Figure 6 represents normalized O-PTIR-spectra of the reference sample and the samples including Joncryl ADR 4400. The results reveal that the intensity of the characteristic PET bands for C=O (1727 cm^–1^), ethylene glycol segment (1341 and 1408 cm^–1^), ester group (1248–1286 and 1122 cm^–1^), as well as for the aromatic ring (1019–1020 cm^–1^) increases with the increase of the additive concentration to 3 wt %. No additional peaks were observed after the use of the additives. Among the characteristic PET bands, the intensity of the carbonyl group at approximately 1727 cm^–1^ shows the highest sensitivity toward the use of CE, i.e., it increases after the use of 0.5 wt % Joncryl and decreases after the use of 1.5 wt % and increases, whereas other bands do not show differences at these concentrations. The intensity band at approximately 1727 cm^–1^ is known to be a measure of the amorphous content of PET.^25^ The DSC results presented in the above section report a slight decrease in crystallinity with the increasing Joncryl content, and the O-PTIR results agree with the DSC results.

**Figure 7:**
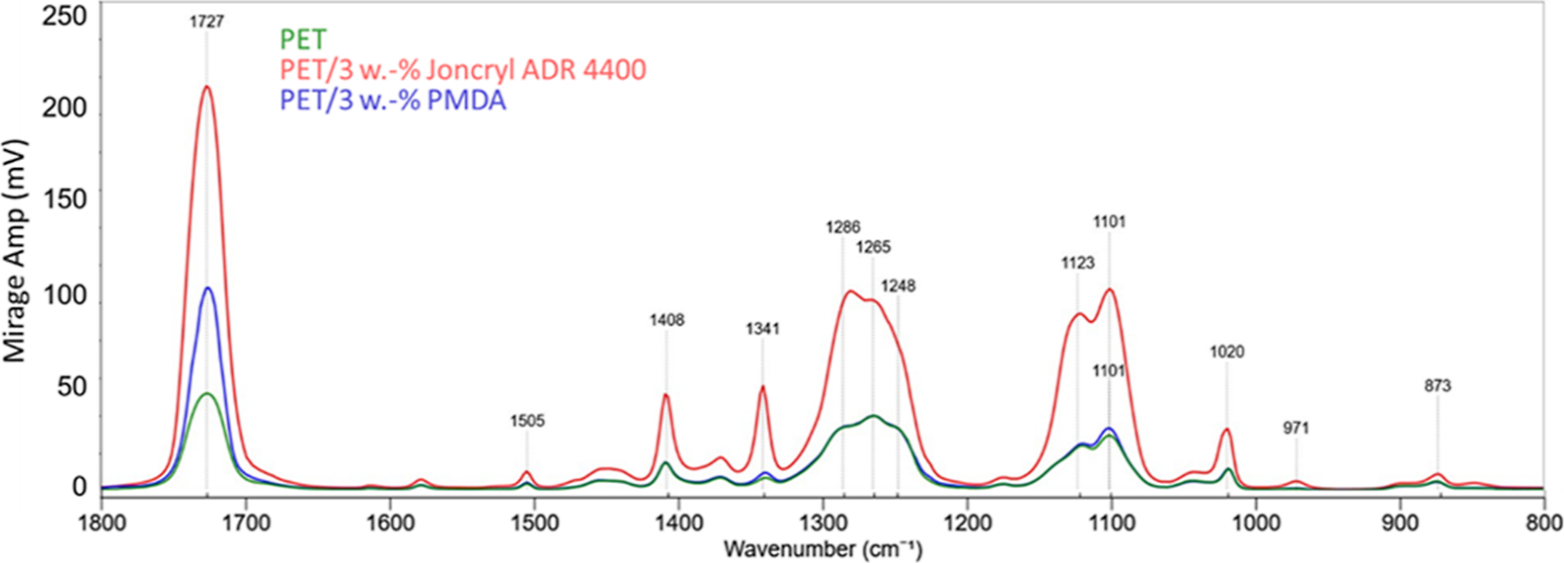
O-PTIR spectra of the samples with 3% of chain extender (Joncryl ADR 4400 and PMDA).

Figure 7 exhibits the O-PTIR spectra of the PET samples with two different CEs as additives. According to these results, the additives have a partly different effect on the intensity of the individual functional groups. Except for the carbonyl band at 1727 cm^–1^, PMDA shows minimal difference regarding the reference PET sample. The increase of the intensity at 1727 cm^–1^ may refer to the increase of the amorphous content at the analyzed sample locations.

### 3.6 Rheology Measurements

**Figure 8:**
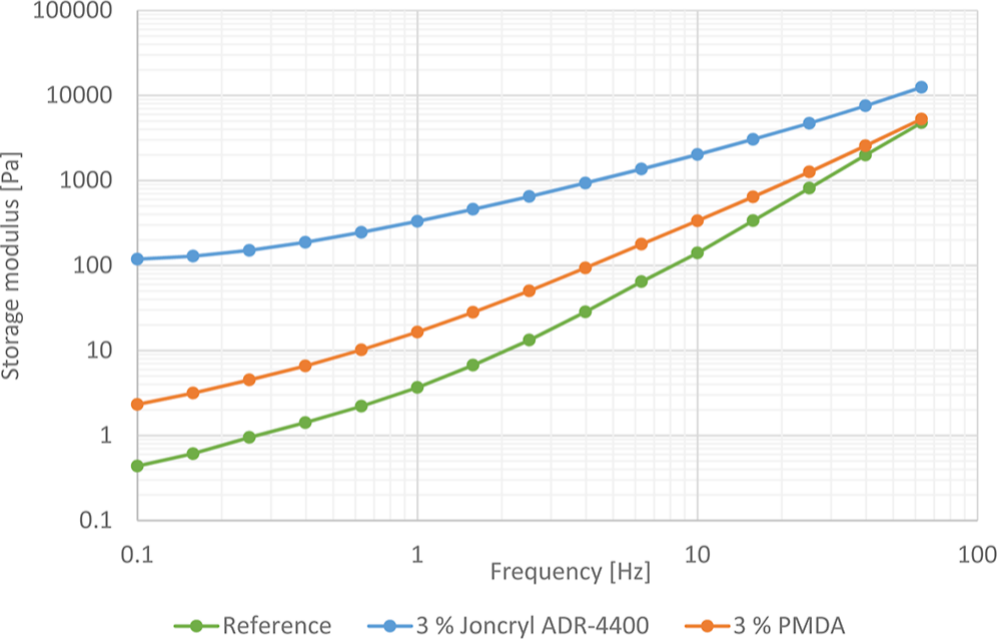
Storage modulus for unmodified PET and PET modified with 3% of different CEs.

**Figure 9:**
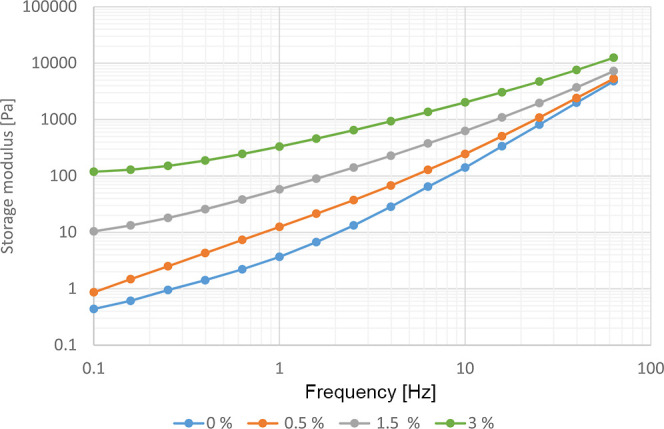
Storage modulus for unmodified PET and PET with different concentrations of Joncryl ADR-4400.

In the literature, various works are mentioned with different concentrations of CEs (up to 3% by weight), especially concerning PMDA and Joncryl,^17,27^ where it has been reported that the molar mass of the recycled PET could be increased.^27^ Since in our case a mixture of recycled PETs from bottles and trays was used and CEs led to improved thermal stability and rheological properties of recycled PET, different concentrations of CEs were considered for rheological investigations. Awaja et al.^18^ pointed out that the chain reactive extrusion process strongly depends on the chain extender PMDA concentration. Figures 8 and 9 show the storage modulus [Pa] as a function of the frequency. At low frequencies, there is enough time for relaxation of the polymer chains, resulting in a low storage modulus. However, at higher frequencies, the polymer chains do not have the opportunity for relaxation, leading to an increased storage modulus. In the case of CE-modified samples, the storage modulus was significantly higher at low frequencies. This indicates an increase in relaxation time and elasticity due to an increase in chain length and entanglement. The storage modulus for the CE Joncryl ADR-4400 is the highest, followed by the CE PMDA, showing that the CE Joncryl ADR-4400 is the most efficient chain extender, forming longer polymer molecules or branching. The storage modulus increases with the increasing Joncryl concentration up to 3 wt %, indicating a gradual increase of the polymer chain length and the formation of a branched molecular structure. Härth and coauthors have concluded from their recent research that the materials with PMDA consist of linear and tree-like molecules and that the materials with Joncryl include linear, highly branched tree-like molecules and gel structures.^30^ The generated water molecules could initiate further hydrolytic reactions which generate more free-radical ends. It seems that the PMDA concentration and the reaction time are the main factors that determine the blocking, coupling, and branching reactions and hence the carboxyl content which could lead to lower storage modulus values.

Figure S6 exhibits the storage and loss modulus as a function of frequency for unmodified PET. Run 2 is done directly after run 1 on the same sample, and a slight increase in both storage and loss modulus is observed for the second run as compared with the first run. This effect has earlier been reported by Chen et al.^37^ and was explained by polycondensation of the PET chains during the measurement. It was verified for dry samples tested under nitrogen atmosphere.

Figure S7 shows the storage modulus for unmodified PET and PET added 3 wt % Joncryl ADR-4400 from two different experiments. The reproducibility is quite good for unmodified PET, but for the sample with 3 wt % Joncryl ADR-4400, it is a large variation of the storage modulus values between the samples from two different batches. The large variation can be attributed to inhomogeneities in the recycled PET, resulting in differences in the reactivity between PET and the chain extender. Furthermore, there could be more low-molecular-weight compounds in one of the samples with 3 wt % Joncryl ADR-4400. Accordingly, the value for the dispersity is relatively high, as can be seen in Table 2.

### 3.7 NMR Investigations

**Figure 10:**
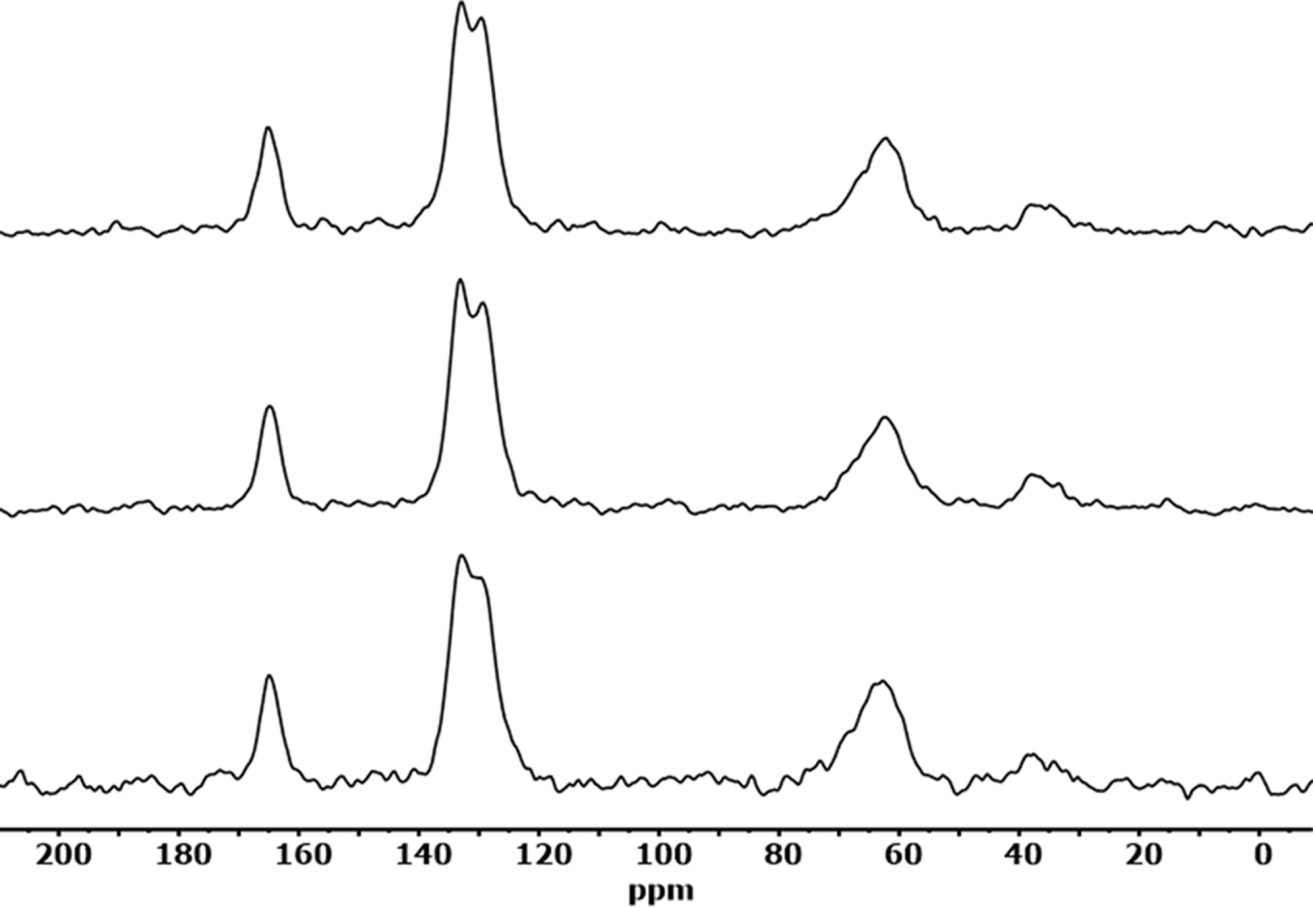
^1^H–^13^C solid-state CP NMR spectra. From bottom to top: PET (no CE), Joncryl ADR-4400, and PMDA.

So far, only a few NMR studies have been performed on recycled PET. These include ^1^H NMR PET end group analysis using derivatization (without chain extension) by Donovan and Moad,^38^ a PET study by Kossentini-Kallel et al.^39^ using ^13^C NMR spectroscopy and PMDA as CE, and a ^1^H NMR study of PET using tetra-glycidyl diamino diphenylmethane as CE.^40^ Our NMR investigations were carried out on neat PET (without CE) and extruded PET with 3 wt % each of Joncryl ADR-4440 and PMDA as CEs, both as solids (^13^C and of dissolved samples ^1^H). Figure 10 shows the ^1^H–^13^C CP solid-state spectra from each of these.

The major C-backbone in the polymers consists of carbonyl groups, aromatic rings, and C atoms bonded to O atom as expected, observed by peaks at 164.9, 132.9, 122.0, and around 65 ppm, respectively. The small intensity peaks just below 40 ppm are due to the spinning sidebands from the aromatic C atoms. The spectra are rather similar, and this is a strong proof of the fact that the CE reactions have progressed as expected, and no unwanted compounds/structural sections in the modified polymers have been formed. However, there is one notable difference. The peaks around 130 ppm for the two CE samples are slightly more resolved compared to PET with no CE. A peak’s shape (width, shape) in solid-state NMR spectra may often be influenced by effects such as crystallinity and defects with the effect that, e.g., amorphous polymers have broader peaks compared to the more crystalline version of the same polymer. The higher resolved CE sample’s peaks from aromatic carbon atoms might indicate a different large-scale structure/packing toward a more regular/ordered backbone. ^1^H MAS solid-state spectra did not show any appreciable resolution, and we progressed further by dissolving the polymer samples as described in the experimental part.

**Figure 11:**
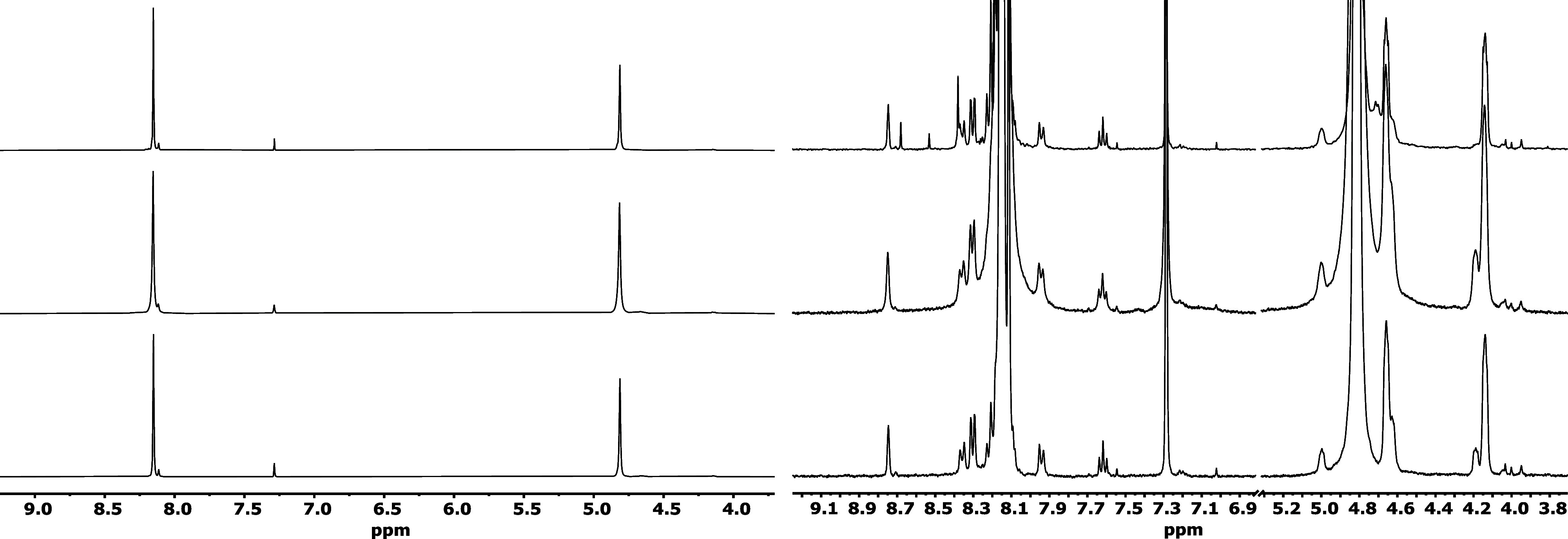
^1^H solution NMR spectra of dissolved PET samples. Left stack overview, right stack zoom-in and magnified intensity. From bottom to top: PET (no CE), Joncryl ADR-4400, and PMDA.

Figure 11 reveals the ^1^H NMR spectra of the three dissolved PET samples which were selected. These are the same as shown in Figure 10. The peak at 8.15 ppm is due to the aromatic protons, and the peak at 4.80 ppm is due to the –CH_2_CH_2_– sections in the polymers. The small visible peak at 7.29 is due to the solvent impurity CHCl_3_. Peaks at 7.61, 8.35, and 8.74 ppm are signs of small amount of iso-PET. In the top spectrum (PMDA), peaks are seen at 8.38, 8.52, and 8.68 ppm and are attributed to protons at the aromatic ring of the added pyromellitic anhydride. This compound originally had only one type of proton, but with the different types of reactions of the end-rings, these protons do become inequivalent. The absence of these peaks in the reference material shows that the origin is from PMDA. In the middle row (Joncryl), formation of −CH_2_CH_2_–O −pCH_2_CH_2_– sections is evidenced by the peaks at 4.13 and 4.65 ppm.^41^ Two more multicomponent peaks located at chemical shifts of 4.19 and 4.62 ppm are due to other variations of these –CH_2_CH_2_– sections.

There are still multiple small peaks in the spectra that presumably can, e.g., be attributed to the unreacted ends of the PET chains, ordering variations, side reaction products, impurities, or variations of sections generated in the chain extension reactions. One peak is found at 1.3 ppm, and together with the peaks mentioned above at ca. 4.2 ppm, this might indicate end groups of the type –OCH_2_CH_3_.

Unreacted epoxy groups should give three peaks in the H spectrum, one for each of the protons on the three-membered ring since these are nonequivalent in an NMR spectrum (see ref (42) for the assignment of Hs in epoxy rings). Since we do not observe three related peaks in the Joncryl sample, around 2.5–3 ppm, we suspect that the CE reaction has gone to completion in the sense that all epoxide chain-ends are consumed. For PMDA, however, we observe three different peaks which indicate that not all four reaction sites have undergone reactions.

## 4 Conclusions

In this article, we have presented analyses of recycled PET through chain extension reaction. The influence of the type and concentration of CEs on the viscosity and other rheological properties was studied. The results from different analysis methods show that chain extension with Joncryl proceeds better compared to the reaction with PMDA.

Measurements of force versus time during extrusion, viscosity, and rheology (storage modulus) result in significantly higher values for the samples with Joncryl as the CE compared to PMDA as the CE. The same analysis methods exhibit an increase in values with increasing amounts of Joncryl. SEC measurements also support that the CE reaction is more effective for Joncryl than for PMDA; for the highest tested CE concentration (3 wt %), a large amount of gel formation was observed with Joncryl as the CE, leading to insoluble products. Liquid NMR measurements on samples with 3 wt % Joncryl indicate that the CE reaction has gone to completion as all epoxide chain ends appear to have been consumed. This is in contrast to the case of the PMDA reaction, where NMR data show that not all four reaction sites have undergone a reaction.

From DSC measurements, it was observed that the crystallinity decreases for the highest amount of added Joncryl, while for 3 wt % PMDA, the crystallinity was on the same level as for unmodified PET, which is also an indication of more effective chain extension with Joncryl as compared with PMDA. Optical photothermal IR reveals that addition of both types of CEs leads to an intensity increase from C=O and C–O bands with a more pronounced increase for Joncryl. The increase in the C=O band can indicate that the amount of amorphous material increases with more added CE, which agrees with the DSC observations.

SEC and gel content analysis have shown changes in molecular mass and molecular mass distribution of the tested samples. Increase of average molecular mass *M*_w_ was recorded in the case of Joncryl CE up to 1.5% concentration. In the case of the sample with 3% Joncryl, formation of high gel content, which had to be filtered out, resulted in decreased *M*_w_ value. An increase in molar mass dispersity up to 1.5 wt % Joncryl concentration was also observed. Both observations lead to the conclusion that the polymer has undergone reactions leading to chain extension. Changes observed in the molecular mass of small-molecular moieties detected in SEC measurements suggest that the introduced Joncryl CE initially reacts with those moieties due to their superior mobility. Only upon exhaustion of those small molecules, the chain extension of PET can advance. This suggests that a higher-than-usual concentration of CE should be used in cases where the compound can be contaminated, e.g., in the case of recycled PET, to compensate for the loss of CE engaged in reaction with contaminants.

From the results of Py–GC/MS, it can be concluded that for Joncryl ADR 4400, the styrene peak and the styrene trimer can serve as fingerprints for this chain extension additive. Moreover, the styrene signal intensity is proportional to the concentration of the additive, which allows for the calculation of CE concentration in the compound. Additionally, detection of a quasi-PET trimer could be used as an indication for CE utilization. The PMDA presence was detected in thermodesorption mode, which indicates that at least a part of this CE has not participated in the cross-linking reaction, which is in line with NMR data.

Overall, the physical properties of the CE PET polymers were enhanced, and the presented improvement of recycled plastics is a viable path to progress on for circular processes. We think that our proposed method setup could be a suitable way to characterize recycled PET samples from different sources.

## Supporting Information Available

Example of a DSC thermogram for PET with chain extender; chromatograms of TIC for reference sample and different concentrations of Joncryl ADR-4400; plot of thermodesorption measurements on the reference sample and different concentrations of Joncryl ADR-4400; pyrogram of sample containing PMDA; O-PTIR spectrum of reference PET; plot of storage and loss modulus for unmodified PET; and plot of storage modulus for unmodified PET and PET with 3% Joncryl ADR-4400 from two different batches (PDF)
