# Supplementary material for: Upgrading and Enhancement of Recycled Polyethylene Terephthalate with Chain Extenders: In-Depth Material Characterization
Source: Ind Eng Chem Res. 2024 Jul 5;63(28):12277–87. doi: 10.1021/acs.iecr.4c00018 (PMC11261598; doi:10.1021/acs.iecr.4c00018)
Supplement: Supplementary file 1 — ie4c00018_si_001.pdf [file ie4c00018_si_001.pdf]

Supporting information

Upgrading and enhancement of recycled PET with chain extenders: In-depth material characterization

Christian W. Karl[1], Bjørnar Arstad[2], Madina Shamsuyeva[3], Jacek Lecinski[3], Kjell Olafsen[1], Åge Gellein Larsen[1], Stephan Kubowicz[1], James Comerford[2] and Hans-Josef Endres[3]*

*(Corresponding author: christian.karl@sintef.no)

[1] SINTEF Materials and Nanotechnology, Polymer and Composite Materials Group, PO box 124 Blindern, 0314 Oslo, Norway

[2] SINTEF Process Technology, Process Chemistry and Functional Materials Group, PO Box 124, 0314 Blindern, Oslo, Norway

[3] IKK - Institute of Plastics and Circular Economy, Leibniz Universität Hannover, An der Universität 2, 30823 Garbsen, Germany

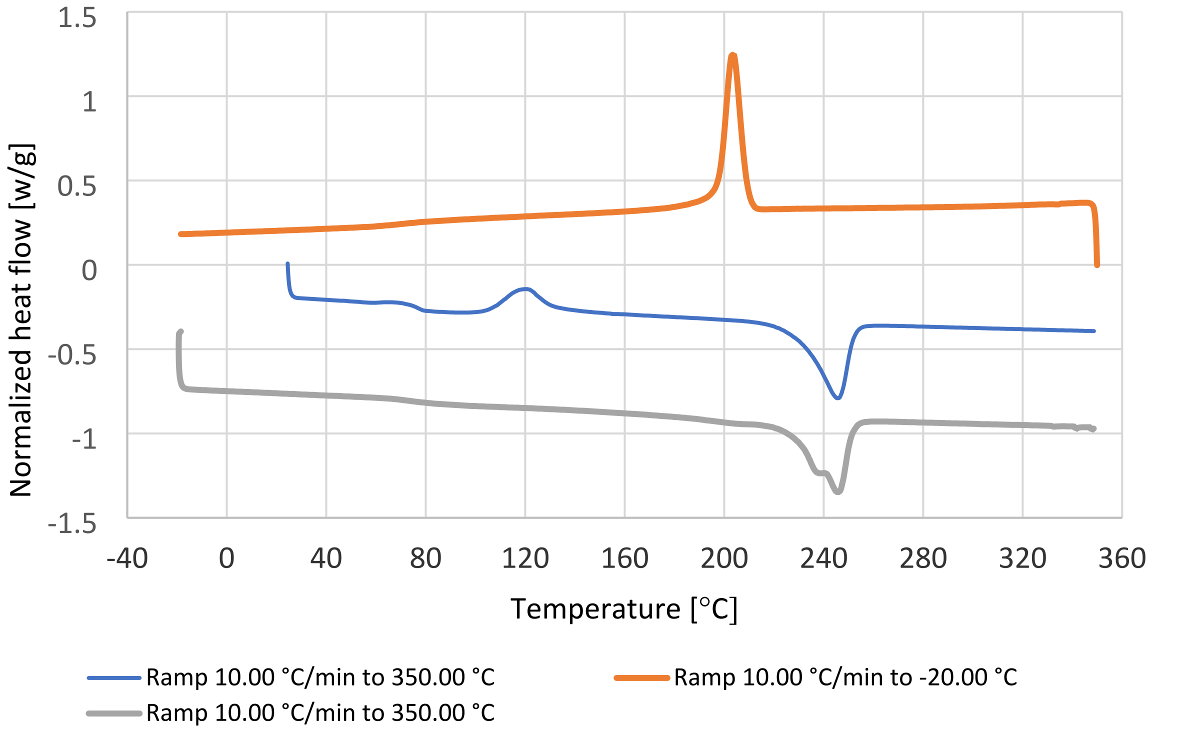

Figure S1 An example of a DSC thermogram for PET with chain extender (3 wt.-% Joncryl® ADR-4400) showing first heating, first cooling down and second heating.

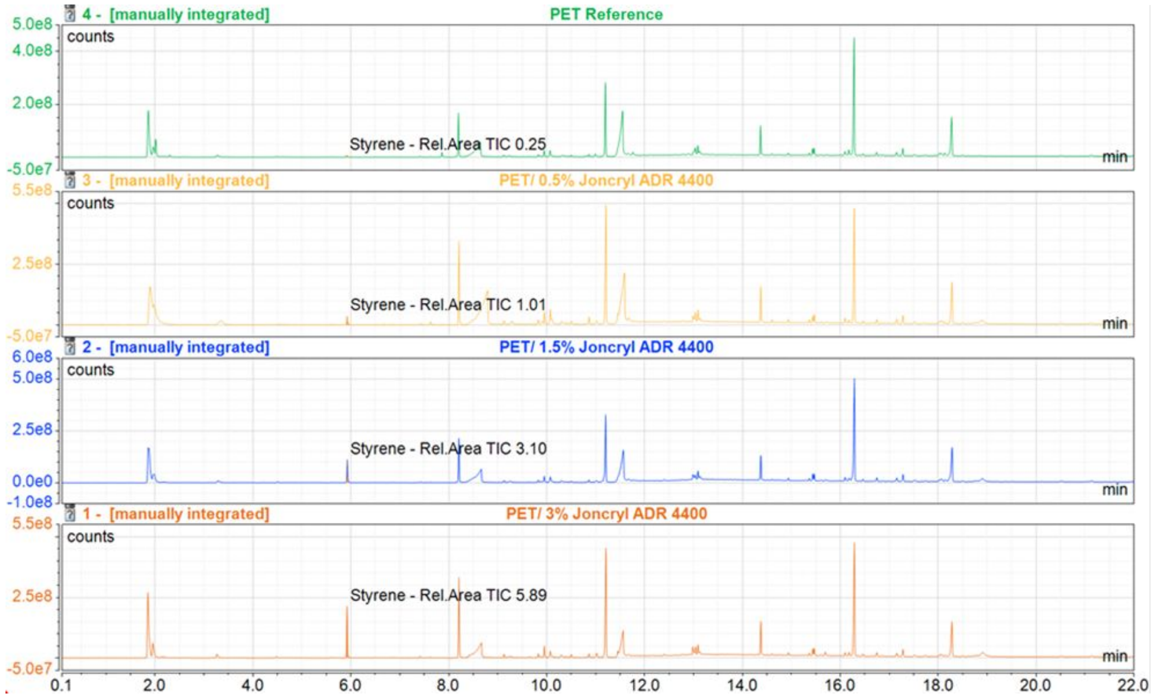

Figure S2 Chromatograms of Total Ion Current for the reference sample (top) and samples with different concentrations of Joncryl® ADR 4400 detected only in compounds using those additives and not in the reference (bottom)

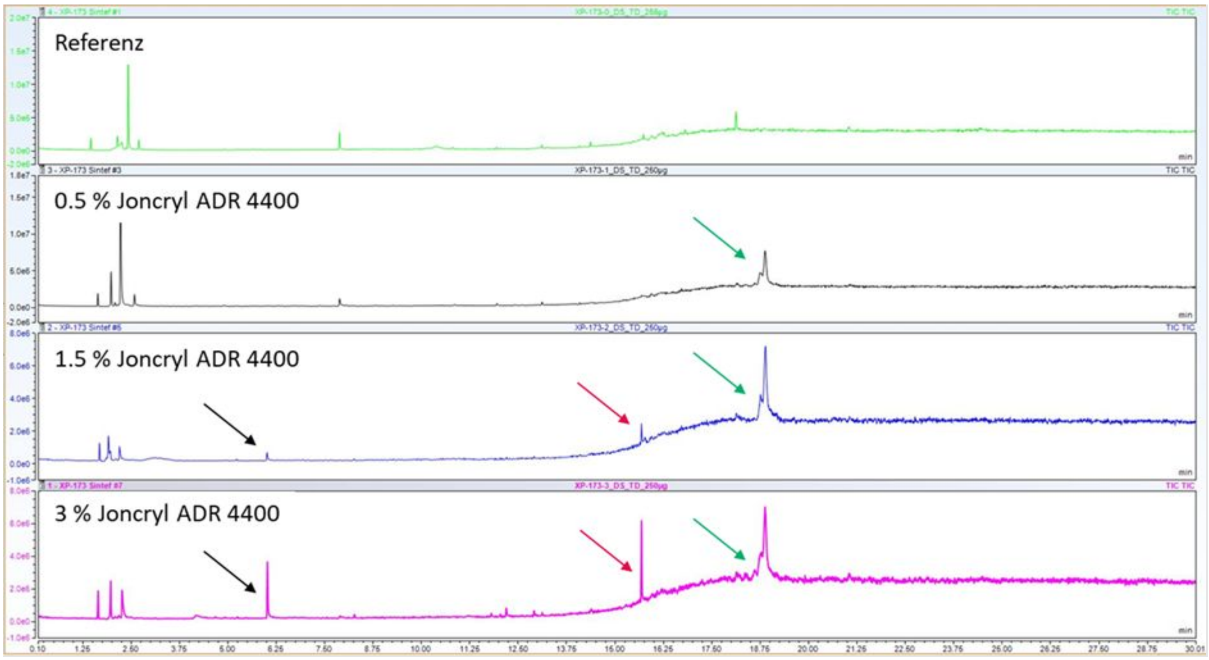

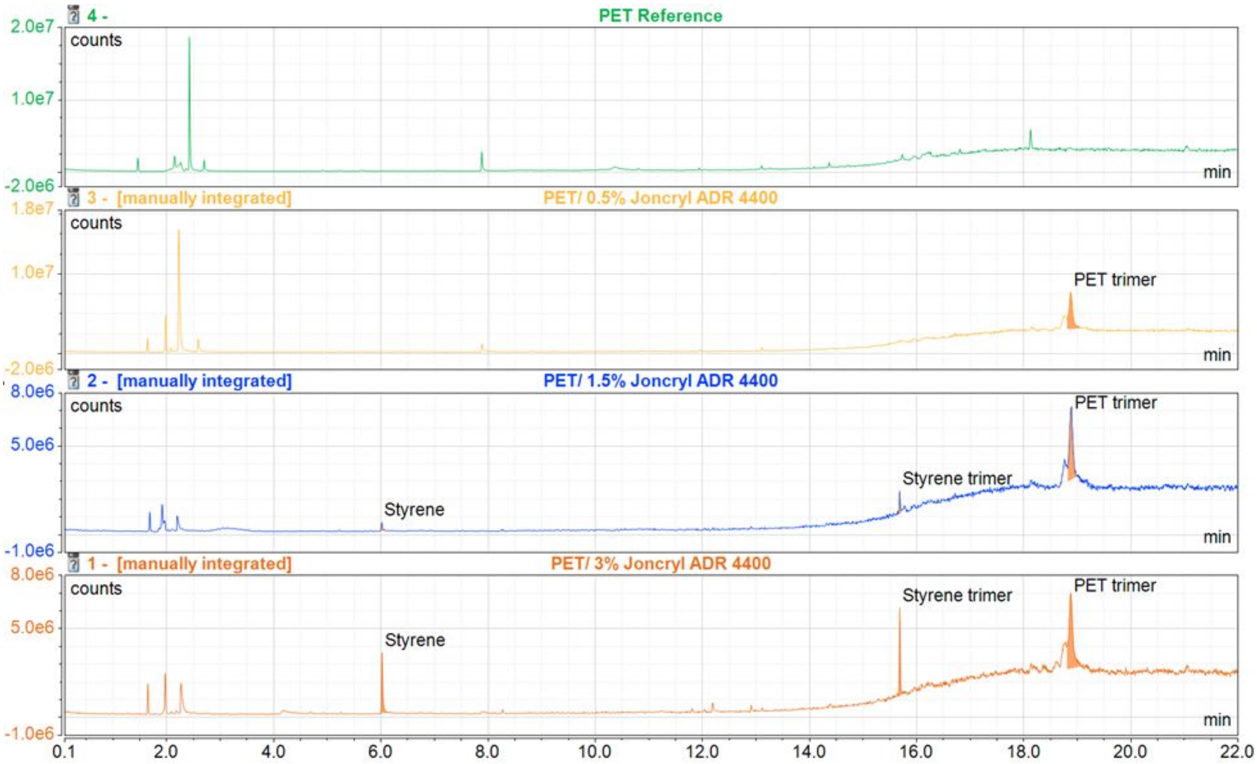

Figure S3 Results of thermodesorption measurements of the reference and samples with Joncryl® ADR 4400.

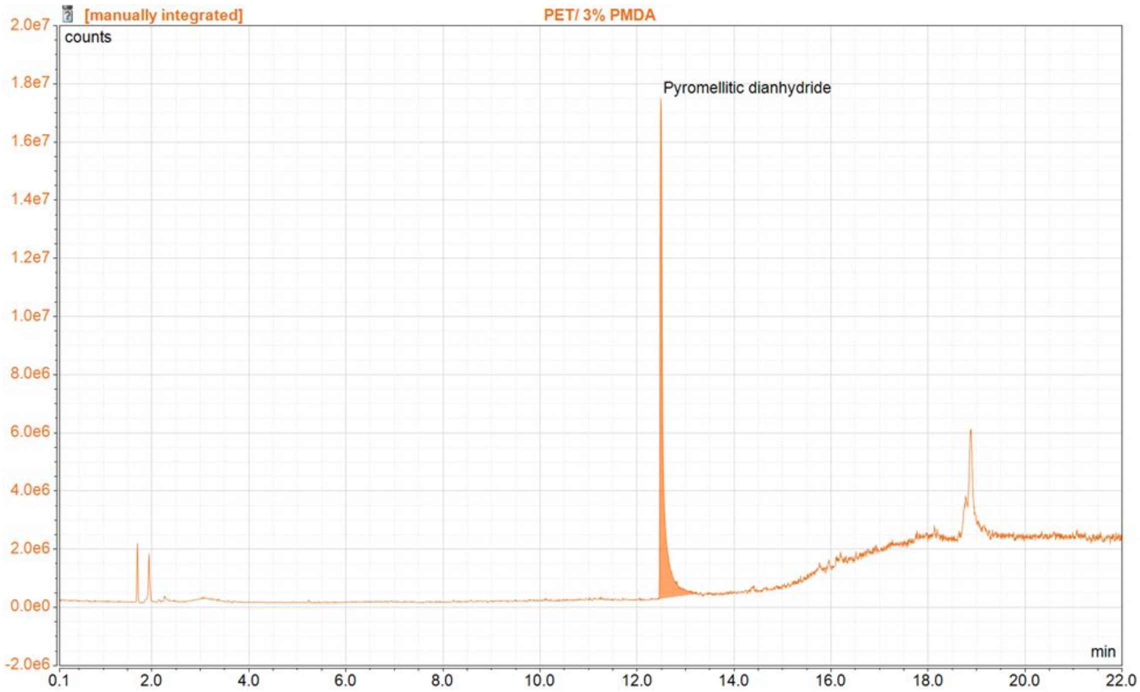

Figure S4 Pyrogram of the sample containing PMDA.

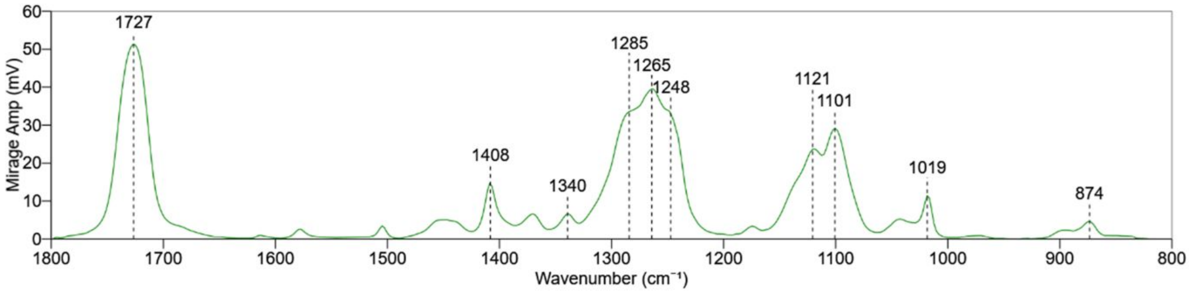

Figure S5 O-PTIR spectrum of the reference PET without added chain extender.

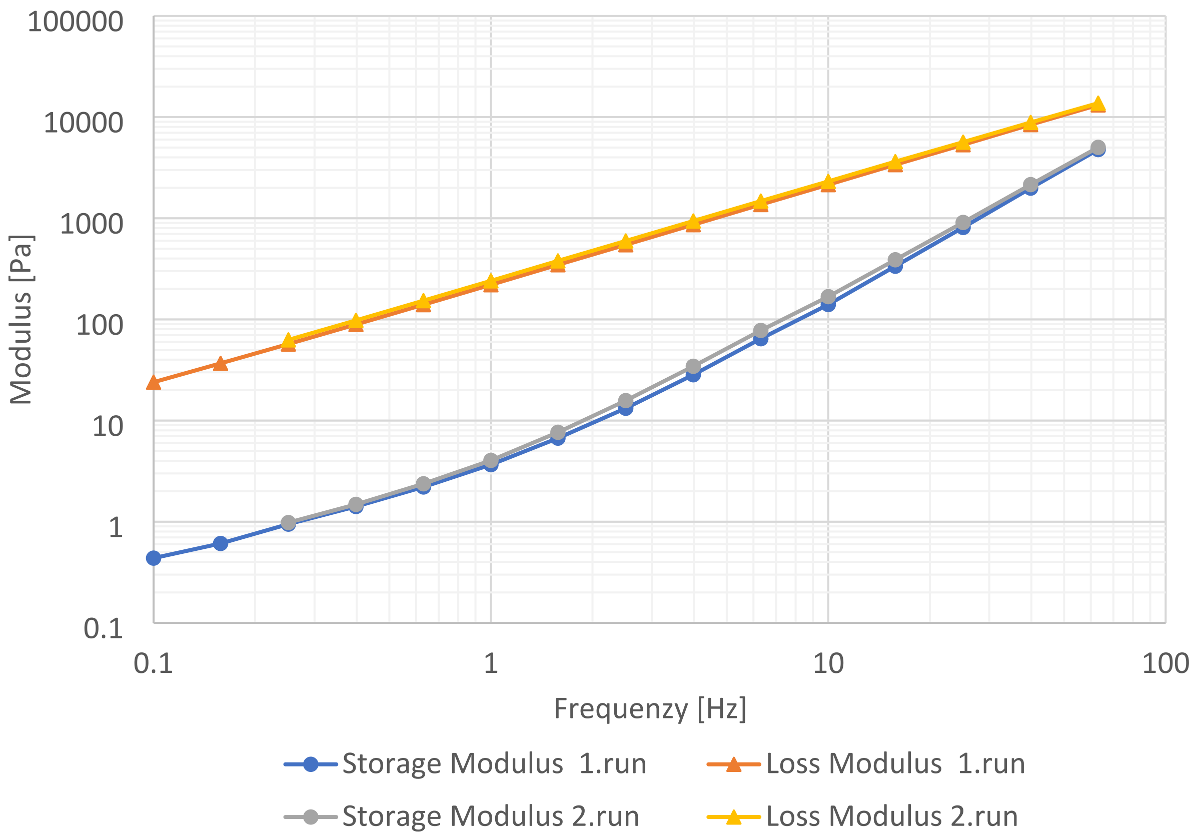

Figure S6 Storage and loss modulus for unmodified PET (no CE) for two runs directly after each other.

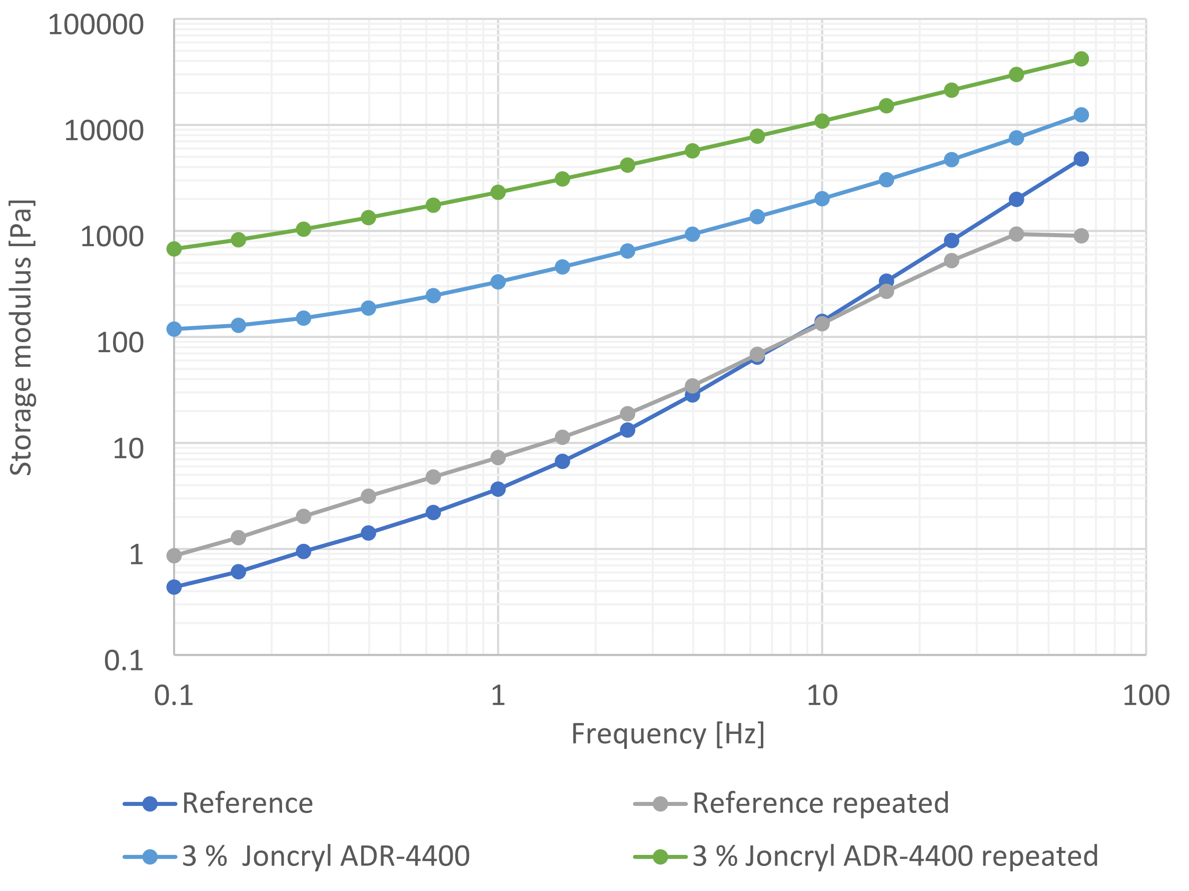

Figure S7 Storage modulus for unmodified PET and PET with 3 % Joncryl® ADR-4400 from two different batches for test of reproducibility.
